# Two Introductory Letters, Delivered by Dr. William Hunter, to His Last Course of Anatomical Lectures, at His Theatre in Windmill Street; as They Were Left Corrected for the Press by Himself. To Which Are Added, Some Papers Relating to Dr. Hunter's Intended Plan for Establishing a Museum in London, for the Improvement of Anatomy, Surgery, and Physic

**Published:** 1785

**Authors:** 


IV. *Two Introductory Letters, delivered by Dr.* William Hunter, *to his laſt Courſe of Anatomical Lectures, at his Theatre in Windmill Street; as they were left corrected for the Preſs by himſelf. To which are added, ſome Papers relating to Dr. Hunter's intended Plan for eſtabliſhing a Muſeum in London, for the Improvement of Anatomy, Surgery, and Phyſic.* 4to. Johnſon, *London,* 1784. 130 pages. 6s. *in Boards.*

IN the firſt of theſe two excellent lectures, the author gives a general ſketch of the origin and progreſs of Anatomy. In tracing the great revolution of learning which happened in the fifteenth century, he has been enabled to carry the the hiſtory of the improvement of anatomy farther back than has been generally done by our own writers; and to introduce into the annals of our art, a genius of the firſt rate, Leonardo da Vinci, who has been overlooked, becauſe he was of another profeſſion, and becauſe he publiſhed nothing upon the ſubject. Dr. Hunter is of opinion, that he was, by far, the beſt anatomiſt and phyſiologiſt of his time, and that his maſter and he were the very firſt who raiſed a ſpirit for anatomical ſtudy, and gave it credit; and Leonardo, he adds, was certainly the firſt man we know of who introduced the practice of making anatomical drawings.

Vaſſare, in his Lives of the Painters, ſpeaking of Leonardo, obſerves, that he "made a "book of ſtudies, drawn with red chalk, and "touched with a pen, with great diligence, of "ſuch ſubjects as he had himſelf diſſected; "where he made all the bones, and to thoſe he "joined, in their order, all the nerves, and "covered them with the muſcles; and con-"cerning thoſe, from part to part, he wrote "remarks in letters of an ugly form, which "are written by the left hand backwards, and "not to be underſtood but by thoſe who know "the method of reading them; for they are

"not

" not to be read without a looking-glafs."— Thefe very drawings and the writing are happily found to be preferved in his Majefty's great collection of original drawings, where Doctor Hunter procured leave to examine them.— They form one large and thick folio, in old Morocco binding, with the following infcription, printed in gold capitals, on each fide of the cover:

DISEGNI DI LEONARDO DA VINCI
RESTAVRATI DA POMPEIO LEONI.

This infcription, as our author obferves, authenticates the collection; for in a note upon the life of Leonardo, Du Pile tells us, from a Latin manufcript of Rubens, then in his hands, among other things, what follows, " Rubens " enlarges on Leonardo's fkill in anatomy. He " adds a particular relation of his ftudies, and " of all the defigns which he made, which Ru- " bens had feen among the curiofities of Pom- " peio Leoni, at Arezzo, who had all his de- " figns." In examining thefe drawings, Dr. Hunter expected to fee little more than fuch defigns in anatomy, as might be ufeful to a painter in his own profeffion. " But I faw (fays " he) and indeed with aftonifhment, that Leo-

" nardo

"nardo had been a general and a deep ſtudent.

"When I conſider what pains he has taken

"upon every part of the body, the ſuperiority

"of his univerſal genius, his particular excel-

"lence in mechanics and hydraulics; and the

"attention with which ſuch a man would ex-

"amine and ſee objects which he was to draw,

"I am fully perſuaded that Leonardo was the

"beſt anatomiſt, at that time, in the world.

"We muſt give the fifteenth century the credit

"of Leonardo's anatomical ſtudies, as he was

"fifty-five years of age at the cloſe of that

"century." Dr. Hunter entertained thoughts of engraving and publiſhing the principal of Leonardo's anatomical deſigns. They would certainly be a curious acquiſition to the hiſtory of anatomy.

The veneration with which we have always been accuſtomed to contemplate the character of our illuſtrious countryman Harvey, and his diſcovery of the circulation, makes us wiſh that the following paſſage had been omitted, where Dr. Hunter, in treating of this part of the hiſtory of Anatomy, ſays, "So much had been

"diſcovered by others, that little more was

"left for him to do, than to dreſs it up into

"a ſyſtem; and *that*, every judge in ſuch mat-

" ters will allow, required no extraordinary ta-
" lents. Yet, eafy as it was, it made him *im-*
" *mortal.* But none of his writings fhew him
" to have been a man of uncommon abilities.
" It were eafy to quote many paffages, which
" bring him nearly to a level with the reft of
" mankind. He lived almoft thirty years after
" Afellius publifhed the Lacteals, yet, to the
" laft, feemed moft inclined to think, that no
" fuch veffels exifted. Thirty hours, at any
" time, fhould have been fufficient to remove
" all his doubts."

After having confidered the rife and progrefs of anatomy; the various difcoveries that have been made in it, from time to time; and the great number of diligent obfervers who have applied themfelves to this art, Dr. Hunter proceeds to point out the importance of this ftudy to the phyfician and furgeon; and here he takes occafion to defcribe the advantages arifing from the diffection of morbid bodies. " Were I to guefs (fays he) at the moft pro-
" bable future improvements in phyfic, I fhould
" fay, that they would arife from a more gene-
" ral, and more accurate examination of dif-
" eafes after death; and were I to place a man
" of proper talents in the moft direct road for
" becoming

" becoming truly *great* in his profeſſion, I

" would chuſe a good practical anatomiſt, and

" put him into a large hoſpital to attend the

" ſick, and diſſect the dead."

That beginners in the ſtudy of anatomy may acquire a ſatisfactory general idea of their ſubject, he ſuppoſes the mind, or immaterial part, to be placed in a corporeal fabric, to hold a correſpondence with other material beings, by the intervention of the body, and then conſiders, *a priori*, what will be wanted for her accommodation. This leads him to deſcribe, in a very pleaſing and intereſting manner, the neceſſity or advantage, and therefore, the final cauſe of moſt of the parts which we actually find in the human body. He begins with pointing out the neceſſity of a brain, or place of immediate reſidence for the mind; of organs of ſenſe, which may enable her to hold a correſpondence with all the material beings which ſurround her; and of nerves, or organs of communication, between herſelf, in the brain, and thoſe organs of ſenſe, to give her information of all the impreſſions that are made upon them, and to convey her commands and influence over the whole. Farther, the mind, in this corporeal ſyſtem, it is obſerved, muſt be endued with the power of moving from place to place, that ſhe may have intercourſe with a variety of objects; that ſhe may fly from ſuch as are diſagreeable, dangerous, or hurtful, and purſue ſuch as are pleaſant or uſeful to her. And accordingly, ſhe is furniſhed with limbs, and with muſcles and tendons, the inſtruments of motion. "But to ſupport, to give firmneſs "and ſhape to the fabric; to keep the ſofter "parts in their proper places; to give fixed "points for, and the proper direction to its "motions, as well as to protect ſome of the "more important and tender organs from "external injuries, there muſt be ſome firm "prop-work (our author obſerves) interwoven "through the whole. And in fact, for ſuch "purpoſes the bones are given. But the prop-"work muſt not be made into one rigid fabric, "for that would prevent motion. Therefore "there are a number of bones.

"Theſe pieces muſt all be firmly bound to-"gether, to prevent their diſlocation. And, in "fact, this end is perfectly well anſwered by "the ligaments.

"The extremities of theſe bony pieces, where "they move, and rub upon one another, muſt "have ſmooth and ſlippery ſurfaces, for eaſy

"motion.

" motion. This is moſt happily provided for, " by the cartilages and mucus of the joints.

" The interſtices of all theſe parts muſt be " filled up with ſome ſoft and ductile matter, " which ſhall keep them in their places, unite " them, and at the ſame time allow them to " move a little upon one another. This end is " accordingly anſwered by the cellular mem- " brane, or adipoſe ſubſtance.

" There muſt be an outward covering over " the whole apparatus, both to give it a firm " compactneſs, and to defend it from a thou- " ſand injuries; which, in fact, are the very " purpoſes of the ſkin, and other integuments.

" And, as ſhe is made for ſociety, and inter- " courſe with beings of her own kind, ſhe muſt " be endued with powers of expreſſing and com- " municating her thoughts, by ſome ſenſible " marks or ſigns; which ſhall be both eaſy to " herſelf, and admit of great variety. And, " accordingly, ſhe is provided with the organs " and faculty of ſpeech; by which ſhe can " throw out ſigns with amazing facility, and " vary them without end.

" Thus we have built up an animal body " which would ſeem to be pretty compleat. " But we have not yet made any proviſion for

" its

" its duration. And, as it is the nature of " matter to be altered, and worked upon by " matter; so, in a very little time, such a liv- " ing creature must be destroyed, if there is " no provision for repairing the injuries which " she must commit upon herself, and the inju- " ries which she must be exposed to from with- " out. Therefore, a treasure of blood is actu- " ally provided in the heart and vascular system, " full of nutritious and healing particles, fluid " enough to penetrate into the minutest parts " of the animal; impelled by the heart, and " conveyed by the arteries, it washes every " part, builds up what was broken down, and " sweeps away the old and useless materials. " Hence we see the necessity, or advantage of " the heart and arterial system.

" What more there is of this blood, than " enough to repair the damages of the machine, " must not be lost, but should be returned again " to the heart; and for this purpose the venal " system is actually provided. These requisites " in the animal, explain, *a priori*, the circula- " tion of the blood.

" The old materials which were become use- " less, and are swept off by the current of blood, " must be separated and thrown out of the

" system.

" syſtem. Therefore glands, the organs of ſe-

" cretion, are given, for ſtraining whatever is

" redundant, vapid, or noxious, from the maſs

" of blood; and when ſtrained, they are thrown

" out by emunctories, called excretories.

" Now, as the fabric muſt be conſtantly

" wearing, the reparation muſt be carried on

" without intermiſſion, and the ſtrainers muſt

" always be employed. Therefore, there is

" actually a perpetual circulation of the blood,

" and the ſecretions are always going on.

" But even all this proviſion would not be

" ſufficient; for that ſtore of blood would ſoon

" be conſumed, and the fabric would break

" down, if there were not a proviſion made for

" freſh ſupplies. Theſe we obſerve, in fact,

" are profuſely ſcattered round her, in the ani-

" mal and vegetable kingdoms; and ſhe is pro-

" vided with hands, the fineſt inſtruments that

" could have been contrived for gathering them,

" and for preparing them in a variety of diffe-

" rent ways for the mouth.

" Theſe ſupplies, which we call food, muſt

" be conſiderably changed; they muſt be con-

" verted into blood. Therefore, ſhe is pro-

" vided with teeth for cutting and bruiſing the

" food, and with a ſtomach for melting it

" down;

“ down: in ſhort, with all the organs ſubſer-

“ vient to digeſtion. The finer parts of the

“ aliments only, can be uſeful in the conſtitu-

“ tion: theſe muſt be taken up, and conveyed

“ into the blood, and the dregs muſt be thrown

“ off. With this view the inteſtinal canal is

“ actually given. It ſeparates the nutritious

“ part, which we call chyle, to be conveyed

“ into the blood, by the ſyſtem of abſorbent

“ veſſels; and the fæces paſs downwards, to be

“ conducted out of the body.

“ Now we have got our animal not only

“ furniſhed with what is wanted for its imme-

“ diate exiſtence; but alſo, with the powers of

“ ſpinning out that exiſtence to an indefinite

“ length of time. But its duration, we may

“ preſume, muſt neceſſarily be limited: for as

“ it is nouriſhed, grows, and is raiſed up to

“ its full ſtrength and utmoſt perfection, ſo it

“ muſt, in time, in common with all material

“ beings, begin to decay; and then hurry on to

“ final ruin. Hence we ſee the neceſſity of a

“ ſcheme for renovation. Accordingly, wiſe

“ Providence, to perpetuate, as well as pre-

“ ſerve his work, beſides giving a ſtrong ap-

“ petite for life and ſelf-preſervation, has made

“ animals, male and female, and given them

“ ſuch

" organs and paſſions, as will ſecure the propa-

" gation of the ſpecies, to the end of the

" world.

" Thus we ſee, that by the very imperfect

" ſurvey which human reaſon is able to take of

" this ſubject, the animal man muſt neceſſarily

" be complex in his corporeal ſyſtem, and in its

" operations.

" He muſt have one great and general ſyſtem,

" the vaſcular, branching through the whole,

" for circulation. Another, the nervous, with

" its appendages, the organs of ſenſe, for every

" kind of feeling. And, a third, for the

" union and connection of all thoſe parts.

" Beſides theſe primary and general ſyſtems,

" he requires others, which may be more local

" or confined; one for ſtrength, ſupport, and

" protection; the bony compages: another for

" the requiſite motions of the parts among them-

" ſelves, as well as for moving from place to

" place; the muſcular part of the body: ano-

" ther to prepare nouriſhment for the daily re-

" cruit of the body; the digeſtive organs: and

" one for propagating the ſpecies; the organs

" of generation.

" And, in taking this general ſurvey of what

" would appear, *a priori*, to be neceſſary for

" adapting an animal to the ſituations of huma-

" nity, we obſerve, with great ſatisfaction, that

" man is accordingly, in fact, made of ſuch

" ſyſtems, and for ſuch purpoſes. He has them

" all; and he has nothing more, except the

" organs of reſpiration. Breathing we cannot

" account for, *a priori*; we only know that

" it is, in fact, eſſential and neceſſary to life.

" Notwithſtanding this, when we ſee all the

" other parts of the body, and their functions,

" ſo well accounted for, and ſo wiſely adapted

" to their ſeveral purpoſes, we cannot doubt that

" reſpiration is ſo likewiſe. And if ever we

" ſhould be happy enough to find out clearly

" the object of this function, we ſhall, doubt-

" leſs, as clearly ſee, that the organs are wiſely

" contrived for an important office, as we now

" ſee the purpoſe and importance of the heart,

" and vaſcular ſyſtem; which till the circulation

" of the blood was diſcovered, was wholly con-

" cealed from us.

" The uſe and neceſſity of all the different

" ſyſtems in a man's body, is not more appa-

" rent, than the wiſdom and contrivance which

" has been exerted in putting them all into the

" moſt compact and convenient form; and in

" diſpoſing them ſo, that they ſhall mutually

" receive

"receive, and give helps to one another; and

"that all, or many of the parts, ſhall not only

"anſwer their principal end or purpoſe, but

"operate ſuccesſfully and uſefully, in many

"ſecondary ways.

"If we underſtand and conſider the whole

"animal machine in this light, and compare it

"with any machine, in which human art has

"exerted its utmoſt, ſuppoſe the beſt con-

"ſtructed ſhip that ever was built; we ſhall be

"convinced, beyond the poſſibility of doubt,

"that there is intelligence and power, far

"ſurpaſſing what humanity can boaſt of.

"In making ſuch a compariſon, there is a

"peculiarity and ſuperiority in the natural ma-

"chine, which cannot eſcape obſervation. It

"is this: in machines of human contrivance

"or art, there is no internal power, no prin-

"ciple in the machine itſelf, by which it can

"alter and accommodate itſelf to any injury

"which it may ſuffer; or, make up any in-

"jury which is reparable. But in the natural

"machine, the animal body, this is moſt won-

"derfully provided for, by internal powers in

"the machine itſelf; many of which are not

"more certain and obvious in their effects, than

"they are above all human comprehenſion, as

" to the manner and means of their operation. " Thus, a wound heals up of itſelf; a broken " bone is made firm again by a callus; a dead " part is ſeparated and thrown off; noxious " juices are driven out by ſome of the emunc- " tories; a redundancy is removed by ſome " ſpontaneous bleeding; a bleeding naturally " ſtops of itſelf; and a great loſs of blood, " from any cauſe, is, in ſome meaſure com- " penſated, by a contracting power in the vaſ- " cular ſyſtem, which accommodates the ca- " pacity of the veſſels to the quantity contained. " The ſtomach gives information when the ſup- " plies have been expended; repreſents, with " great exactneſs, the quantity and the quality " of what is wanted in the preſent ſtate of the " machine; and, in proportion as ſhe meets " with neglect, riſes in her demand, urges her " petition with a louder voice, and with more " forcible arguments; for its protection, an " animal body reſiſts heat and cold in a very " wonderful manner, and preſerves an equal " temperature, in a burning and in a freezing " atmoſphere.

" There is a farther excellence, or ſuperi- " ority in the natural machine, if poſſible, ſtill " more aſtoniſhing, more beyond all human

" compre-

"comprehenſion, than what we have been "ſpeaking of. Beſides thoſe internal powers "of ſelf-preſervation in each individual, when "two of them co-operate, or act in concert, "they are endued with powers of making other "animals, or machines like themſelves, which "again are poſſeſſed of the ſame powers of pro- "ducing others, and ſo of multiplying the ſpe- "cies without end.

"Theſe are powers which mock all human "invention or imitation. They are characte- "riſtics of the divine architect."

Having premiſed this general account of his ſubject, Dr. Hunter next conſiders the method to be obſerved in ſtudying Anatomy, and concludes with deſcribing the plan of his own courſe. In doing this, he not only ſets forth what he propoſes to do on his own part, but likewiſe what he expects of his hearers, and his obſervations on this head are admirably calculated to fix the attention of young minds, and to excite in them a ſpirit of diligence and ardour in their anatomical purſuits.

Annexed to the Lectures are ſeveral papers relating to Dr. Hunter's intended plan for eſtabliſhing an anatomical ſchool in London, under the auſpices of Government. In a memorial on this ſubject, Dr. Hunter requeſted only a proper piece of ground, on which he might expend ſix or ſeven thouſand pounds, of his own money, in erecting a building fit for the purpoſe. This national and diſintereſted ſcheme was neglected by Mr. George Grenville, to whom it had been recommended by the Earl of Bute, and after ſeveral months, finding that nothing was likely to be done in the buſineſs, Dr. Hunter, in a ſpirited letter to Mr. Grenville, after begging pardon for having given ſo much trouble, requeſted that he might be no longer conſidered as bound in honour to fulfil his part of the propoſal.

